# Prospective evaluation of serum tissue inhibitor of metalloproteinase 1 and carbonic anhydrase IX in correlation to circulating tumor cells in patients with metastatic breast cancer

**DOI:** 10.1186/bcr2916

**Published:** 2011-07-11

**Authors:** Volkmar Müller, Sabine Riethdorf, Brigitte Rack, Wolfgang Janni, Peter A Fasching, Erich Solomayer, Bahriye Aktas, Sabine Kasimir-Bauer, Julia Zeitz, Klaus Pantel, Tanja Fehm

**Affiliations:** 1 Department of Gynecology, University Medical Center Hamburg-Eppendorf, Martinistrasse 52, 20241 Hamburg, Germany; 2 Hamburg Institute of Tumor Biology, University Medical Center Hamburg-Eppendorf, Hamburg, Germany; 3 Department of Obstetrics and Gynecology, Ludwig Maximilians-University, Lindwurmstraße 2a80337 Munich, Germany; 4 Department of Obstetrics and Gynecology, University Medical Center, Moorenstr. 5, 40225 Düsseldorf, Germany; 5 Department of Obstetrics and Gynecology, University Medical Center, Universitätsstraße 21-2391054 Erlangen, Germany; 6 Department of Obstetrics and Gynecology, University Medical Center, Universitätsklinikum des Saarlandes, Kirrberger Straße, 66424 Homburg/Saar; Germany; 7 Department of Obstetrics and Gynecology, University Medical Center, Hufelandstraße 55, 45147 Essen, Germany; 8 Department of Obstetrics and Gynecology, University Medical Center, Calwerstr. 7, 72076 Tübingen, Germany

## Abstract

**Introduction:**

Circulating tumor cells (CTCs) reflect aggressive tumor behavior by hematogenous tumor cell dissemination. The tissue inhibitor of metalloproteinase 1 (TIMP-1) plays a role in tissue invasion and is also involved in angiogenesis, abrogation of apoptosis and in chemoresistance. Carbonic anhydrase IX (CAIX) is a metalloenzyme involved in cell adhesion, growth and survival of tumor cells. The aim of the study was to investigate whether serum concentrations of TIMP-1 and CAIX are associated with the detection of CTC in metastatic breast cancer.

**Methods:**

Blood was obtained in a prospective multicenter setting from 253 patients with metastatic breast cancer at the time of disease progression. Serum TIMP-1 and CAIX were determined using commercial ELISA-kits (Oncogene Science). CTC were detected with the CellSearch™ system (Veridex).

**Results:**

Five or more CTCs were detected in 122 patients out of 245 evaluable patients (49.8%). Out of 253 metastatic patients 70 (28%) had serum TIMP-1 levels above 454 ng/mL. Serum CAIX was elevated above 506 ng/mL in 90 (35%) patients. Both serum markers had prognostic significance. Median progression free survival (PFS) was 7.2 months with elevated TIMP-1 vs. 11.4 months with non-elevated levels (p < 0.01). OS was 11.5 vs. 19.1 months (p < 0.01). Median PFS was 7.5 months with elevated CAIX vs. 11.7 months with non-elevated levels (p < 0.01), overall survival (OS) was 13.4 months vs. 19.1 months (p < 0.01). In patients with five or more CTCs, serum levels were above the cut-off for CAIX in 47% vs. 25% in those with less than five CTCs (p = 0.01). For TIMP-1, 37% patients with five or more CTCs had elevated serum levels and 17% of patients with less than five CTCs (p = 0.01). Including TIMP-1, CAIX, CTC and established prognostic factors in the multivariate analysis, the presence of CTCs, the therapy line and elevated CAIX remained independent predictors of OS.

**Conclusions:**

Elevated serum levels of the invasion markers TIMP-1 and CAIX in metastatic breast cancer are prognostic markers and are associated with the presence of CTCs. Whether increased secretion of TIMP-1 and/or CAIX might directly contribute to tumor cell dissemination remains to be elucidated in further investigations.

**Trial registration:**

Current Controlled Trials: ISRCTN59722891

## Introduction

In breast cancer patients, hematogenous tumor cell dissemination is a crucial step in tumor progression and blood-borne metastases account for the vast majority of breast cancer-related death. Circulating tumor cells (CTC) derived from primary tumors and metastatic sites can be detected in the circulation. Many methods for the detection of CTC have been described [1,2]. At present, the CellSearch^® ^system, which combines both automated enrichment and immunostaining, is the only standardized technology that was approved by the Food and Drug Administration for the detection of CTC in patients with metastatic breast, colon, and prostate cancer [3-5]. The detection of CTC in blood can provide prognostic information [3,6]. Moreover, CTC detection and characterization has already improved our understanding of the complex process underlying tumor cell dissemination and metastatic progression in breast cancer. It is widely accepted now that the release of tumor cells from solid tumors requires specific mechanisms such as proteolysis and release is enhanced when tumor hypoxia occurs.

The tissue inhibitor of metalloproteinase 1 (TIMP-1) is of interest because it plays a role in tissue invasion and angiogenesis. A negative prognostic impact of serum TIMP-1 as well as tissue protein levels was described in breast cancer, colorectal cancer, and other malignancies [7-9]. At one side, TIMP-1 inhibits matrix metalloproteinases (MMPs) and thus, may influence tumor growth and invasion. On the other side, it has been demonstrated that TIMP-1 may inhibit apoptosis in breast epithelial cells [10-12] and promotes cell growth, tumorigenesis, and angiogenesis in different cell types, including breast carcinoma cell lines [13-15].

Carbonic anhydrase IX (CAIX) is a metalloenzyme involved in cell adhesion, growth, and survival of tumor cells. There is strong evidence that CAIX is involved in tumor cell proliferation as inhibition of CAIX *in vitro *and *in vivo *significantly reduces growth and survival of tumor cells [16]. In several epithelial cancers, CAIX overexpression was shown to be of prognostic relevance [17-21].

Apart from the cellular transmembrane form of CAIX, there is a soluble isoform that is released by proteolytic cleavage and can be detected in peripheral venous blood [22]. Although several reports indicate a role of serum CAIX in renal cell cancer [22,23], information about CAIX in serum of breast cancer patients is limited [24].

In conclusion, several publications demonstrated a biologic role for TIMP-1 in breast cancer whereas information on CAIX is limited. For both markers, experimental and clinical data suggest that they might be also involved in tumor cell dissemination. However, TIMP-1 and CAIX have so far not been examined in combination with CTC measurements as a surrogate marker for hematogenous tumor cell spread. Therefore, the aim of this study was to investigate the role of TIMP1 and CAIX serum levels in association with the presence of CTC in metastatic breast cancer.

## Materials and methods

### Patients

A total of 254 patients with metastatic breast cancer from nine German University Breast Cancer Centers (Düsseldorf (*n *= 4) Erlangen (*n *= 30), Essen (*n *= 46), Freiburg (*n *= 9), Hamburg (*n *= 79), Heidelberg (*n *= 18), Munich (*n *= 16), Regensburg (*n *= 2), and Tübingen (*n *= 50)) were enrolled in this prospective, open-label, non-randomized study. Inclusion criteria were: epithelial invasive carcinoma of the breast with distant metastatic disease (M1), age 18 years and older, and first diagnosis of metastatic disease or disease progression (before start of new treatment regimen). Patients with a second primary malignancy (except *in situ *carcinoma of the cervix or adequately treated cutaneous basal cell carcinoma) were excluded. The primary endpoint of the study was the detection of human epidermal growth factor receptor (HER)2-positive CTC with two different methods [25].

Blood was drawn before the start of a new line of therapy. All patients gave their informed consent for the use of their blood samples. A web-based databank was designed for data management and on-line documentation. By the use of this interface, clinical investigators were blinded for test results and the CTC test sites were blinded for the clinical data of the patients. The study was approved by local institutional review boards (Ethics Board University of Tübingen number 2007/B01) and all patients gave an informed consent. The trial was registered in the Current Controlled Trials Registry (no. ISRCTN59722891).

Patients received systemic therapy according to national and institutional standards. Response was evaluated according to institutional standards usually by computed tomography (CT) scan every 12 weeks. Median follow up from the time point of blood sampling was 11 months (range: 0 to 24 months). At the time of analysis for this study, 172 patients had progressed and 75 patients had died. Patient characteristics are listed in Table 1.

**Table 1:** Patients' characteristics

	Total	CTC positive	in %	*P *value
Overall	245	122	50%	
**ER status**	244	122	50	0.26
Negative	74	33	45	
Positive	170	89	52	
**PR status**	244	122	50	0.51
Negative	99	47	48	
Positive	145	75	52	
**HER2 status**	245	122	123	0.15
Negative^1^	138	76	55	
Positive^2^	75	31	41	
Unknown^3^	32	15	47	
**Metastatic site**	245	122	50	**0.07**
Visceral	96	39	41	
Bone	35	14	40	
Both	114	69	61	
**Extent of metastatic disease**	245	122	50	**0.03**
One site	84	34	41	
Multiple sites	161	88	55	
**Therapeutic setting**	244	122	50	
1st-line	94	48	51	0.48
2nd-line	64	28	44	
3rd-line or more	86	46	54	
**CAIX**	245 (253) ^4^	122	50	
≤506 ng/ml	157 (163)	65	41	**0.00**
> 506 ng/ml	88 (90)	57	65	
**TIMP-1**	245 (253) ^4^	122	50	
≤454 ng/ml	179 (183)	77	43	**0.00**
> 454 ng/ml	66 (70)	45	68	

^1 ^Immunohistochemistry score: 0/+1 or fluorescence in situ hybridization negative, ^2 ^ICH score: +3 or fluorescence in situ hybridization positive

^3 ^not determined or ICH score +2 and fluorescence in situ hybridization not performed

^4 ^in 9 patients CTC could not be determined

CAIX, carbonic anhydrase IX; CTC, circulating tumor cell; ER, estrogen receptor; HER, human epidermal growth factor receptor; PR, progesterone receptor; TIMP, tissue inhibitor of metalloproteinase.

### Enumeration and characterization of CTC

Detection of CTC was performed with the CellSearch assay (Veridex LLC, Raritan, NJ, USA) according to the manufacturers' instructions without modifications. CTC analysis by the CellSearch assay was performed by either of two centers (Institute of Tumor Biology, University Medical Center, Hamburg-Eppendorf (SR, KP) or Department of Gynecology, Munich (BR); 173 and 81 tests, respectively). These centers have previously conducted a validation study demonstrating that samples could be stored and transported (up to 72 hours) as well as examining the high inter- and intra-assay concordance of the results in a multicenter setting [4].

Before the study was started, each breast cancer center was assigned to send its samples only to the designated laboratory for the CellSearch assay. Blood samples for the CellSearch assay were sent at room temperature based on the manufacturer's recommendation. All blood samples were processed within 96 hours for the CellSearch assay, or otherwise discarded. The CellSearch assay was performed by investigators blinded for the clinical data. 7.5 mL blood samples were collected into CellSave tubes (Veridex Inc, Raritan, NJ, USA). The CellSearch Epithelial Cell Test (Veridex Inc, Raritan, NJ, USA) was applied for CTC enrichment and enumeration. In brief, CTC are captured from peripheral blood by anti-epithelial cell adhesion molecule-antibody-bearing ferrofluid and subsequently identified by cytokeratin-positivity/negativity for the leukocyte common antigen CD45 and 4',6-diamidino-2-phenylindole staining to ensure the integrity of the nucleus. A blood sample was considered CTC-positive when at least five CTC were present based on the prognostic relevant cut-off as previously published [3,6].

### Quantitative analysis of serum TIMP-1 levels

Serum TIMP-1 was quantified by commercially available ELISA (Siemens Healthcare Diagnostics, Tarrytown, NY, USA). The serum samples and controls were diluted 1:50 with sample diluent buffer (containing bovine serum albumin, buffer salts and 0.09% sodium azide). A 100 μl volume of the standards, diluted control samples and diluted serum samples were dispensed into 96-well plates (coated with an anti-human monoclonal antibody) and incubated for 30 minutes at room temperature. Wells were washed and 100 μl of the detection antibody (containing alkaline phosphatase-labeled anti-TIMP-1 antibody) were added. Plates were incubated for 30 minutes at room temperature. After washing, 100 μl of chromogenic pNPP-substrate was added for 25 minutes at room temperature in the dark. The reaction was stopped with 100 μl of EDTA-stop solution and absorbance was read at 405 nm by automated plate-reader (Tecan, Crailsheim, Germany). Serum TIMP-1 levels above 454 ng/mL were regarded as elevated as previously described to be of relevance [7].

### Quantitative analysis of serum CAIX level

CAIX was also quantified by a commercially available ELISA (Siemens Healthcare Diagnostics, Tarrytown, NY, USA). Serum samples and controls were diluted 1:2 with sample diluting buffer (containing bovine serum albumin, mouse IgG, buffer salts, and 0.09% sodium azide). One hundred microliters of the standards, of diluted control samples, and of diluted serum samples were dispensed into the wells of a 96-well plate (coated with the monoclonal capture antibody) and incubated for two hours at room temperature on a shaker at 800 rpm. Wells were washed, and 100 μl of the detection antibody (containing biotinylated anti-CAIX antibody) was added. The plates were incubated for 30 minutes at room temperature, washed, and then further incubated with 100 μl of a streptavidin horseradish peroxidase conjugate for 30 minutes at room temperature. After washing, 100 μl of chromogenic substrate (TMB blue substrate) was added for 30 minutes at room temperature. The reaction was stopped with 100 μl of 2.5 N sulphuric acid and absorbance was read at 450 nm by an automated plate reader (Tecan, Crailsheim, Germany). The CAIX concentration was estimated from the standard curve. A value above 506 ng/mL was regarded as elevated (mean of a age matched control + two standard deviations) [24].

The TIMP-1 and CAIX concentrations were estimated from the standard curve. Each sample, standard and control were analyzed in duplicate. Inter-assay and intra-assay coefficients of variation for both serum assays were less than 10%.

### Statistical analysis

Primary endpoint of the analysis described here was the impact of TIMP-1 and CAIX alone and of each marker in correlation to the presence of CTC. The study was performed in accordance with REMARK criteria [26,27]. Relations between categorical variables were investigated using contingency tables. In case of independent data, Fisher's exact test was used to evaluate the relation, whereby *P *values less than 0.05 indicate statistical significance. When paired data were considered in terms of assessing the reliability of test results of the methods, agreement and consistency were regarded via Cohen's kappa and McNemar-test, respectively. The four groups of patients (elevated/non-elevated CTC and serum markers) were compared using the log-rank test to evaluate whether there is a difference in survival. In addition we have also tested for comparing the marker positivity within CTC negative and CTC positive patients. Progression-free survival (PFS) was defined as the time elapsed between blood draw time point and disease progression. Overall survival (OS) was defined as the time elapsed between blood draw time point and patient's death. The Kaplan-Meier curves were compared using log-rank test. Factors which have been of prognostic significance in the univariate analysis were included in a multivariate analysis using the Cox regression model. Statistical analysis was performed using SPSS version 18.

## Results

### Detection rate of CTC and correlation with clinical parameters

Five or more CTC were detected in 122 of 245 evaluable patients (49.8%) and 180 patients (74%) had one or more CTC. Elevated CTC levels of 5 CTC/7.5 mL or more were only associated with the extend of metastatic disease, and elevated TIMP-1 and CAIX levels. The characteristics of patients and correlations with CTC detection are shown in Table 1.

### Serum levels of TIMP-1 and correlation with progression free and overall survival

Of 253 metastatic patients, 70 (28%) had elevated serum TIMP-1 levels above 454 ng/mL. Patients with elevated TIMP-1 levels were more likely to have multiple metastases (*P *< 0.05) located in bone and visceral organs (*P *< 0.01) and had more lines of previous therapy (*P *< 0.01). No other correlations with clinicopathological factors could be observed (data not shown).

Median PFS was 7.2 months in patients with elevated TIMP-1 versus 11.4 months in those with non-elevated levels (*P *< 0.01). OS was 19.1 months vs. 11.5 months (*P *< 0.01). PFS and OS rates are listed in Table 2.

**Table 2:** Mean survival in correlation to CTC positivity, TIMP-1 and CAIX levels

	PFS in months	*P *value	OS in months	*P *value
**CTC**				
< 5 cells	10.9 (9.4-12.5)	0.118	20.1 (18.8-21.5)	< 0.01
≥ 5 cells	9.3 (7.8-10.9)		14.0 (12.8-21.5)	
**TIMP-1**				
Non elevated	11.4 (10.1-12.7)	< 0.01	19.1 (18.0-20.3)	< 0.01
Elevated	7.2 (5.6-8.9)		11.5 (9.4-13.6)	
**CAIX**				
Non elevated	11.7 (10.3-13.1)	< 0.01	19.1 (17.8-20.4)	< 0.01
Elevated	7.5 (6.0-9.0)		13.4 (11.4-15.4)	
**TIMP-1/CAIX**				
Both non-elevated	12.4 (10.9-13.9)	< 0.01	20.1 (18.8-21.3)	< 0.01
Both elevated	7.0 (5.0-9.0)		11.1(8.5-13.6)	

CAIX, carbonic anhydrase IX; CTC, circulating tumor cell; OS, overall survival; PFS, progression-free survival; TIMP, tissue inhibitor of metalloproteinase.

### Serum levels of CAIX and correlation with progression free and overall survival

Serum CAIX was elevated above 506 ng/mL in 90 (35%) patients. Elevated CAIX levels were only correlated with line of therapies (*P *< 0.01). Patients with elevated CAIX-levels were more likely to have visceral metastases.

Median PFS was 7.5 month in patients with elevated CAIX versus 11.7 months in those with non-elevated levels (*P *= 0.001). OS was 13.4 months vs. 19.1 months (*P *< 0.01, see Table 2).

**Figure 1:**
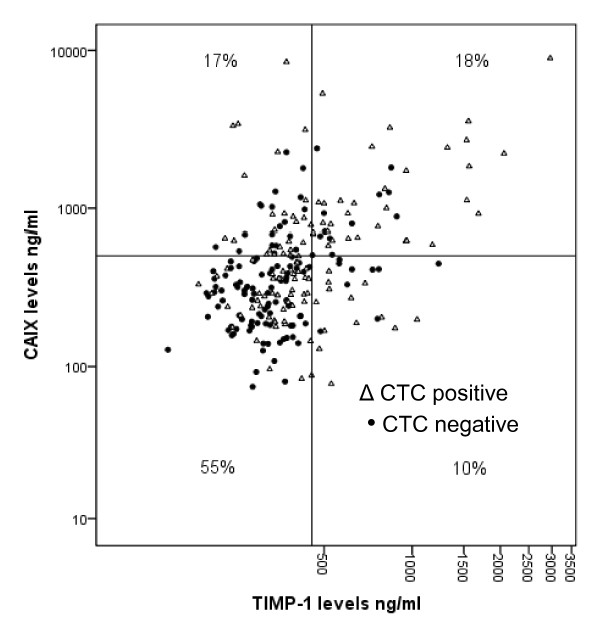
**Correlation between TIMP-levels (ng/ml) and CAIX levels (ng/ml) subdivided after presence of CTC**. CAIX, carbonic anhydrase IX; CTC, circulating tumor cell; TIMP, tissue inhibitor of metalloproteinase.

Elevated levels of both CAIX and TIMP-1 were seen in 18% of patients (Figure 1; Table 2 and 3). When both serum markers were non-elevated the median PFS was 12.4 and OS 20.1 months compared with 7.0 and 11.1 months, respectively, when both serum markers were elevated.

**Table 3:** Serum markers and CTC levels in metastatic breast cancer patients

CAIX/TIMP-1	n (100%)	CTC < 5 cells	CTC > = 5 cells
Total	245 (100%)	123 (100%)	122 (100%)
Both non-elevated	135 (55%)	83 (68%)	52 (43%)
TIMP-1 elevated	66 (27%)	21 (17%)	45 (37%)
CAIX elevated	88 (36%)	31 (25%)	57 (47%)
Both elevated	44 (18%)	12 (10%)	32 (26%)

CAIX, carbonic anhydrase IX; CTC, circulating tumor cell; TIMP, tissue inhibitor of metalloproteinase.

### Correlation of CTC detection to serum levels of TIMP-1 and CAIX

In patients with five or more CTC, serum levels were above the cut-off for CAIX in 47% vs. 25% in those with less than five CTC (*P *= 0.01). For TIMP-1, 37% patients with five or more CTC had elevated serum levels in contrast to 17% with less than five CTC (*P *= 0.01). Correlation between both serum markers and CTC status is illustrated in Figure 1 and summarized in Tables 2 and 3.

### Correlation of CTC detection in combination to serum levels of TIMP-1 and CA IX with progression free and overall survival

**Figure 2:**
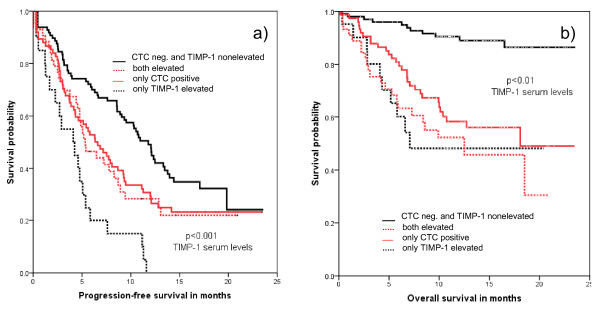
**Correlation between CTC detection, TIMP-1 and patient outcome**. **(a) **Progression-free survival. **(b) **Overall survival of metastatic breast cancer patients based on CTC positivity (≥ 5 CTC) and TIMP-1 serum levels. CTC, circulating tumor cell; TIMP, tissue inhibitor of metalloproteinase.

Among patients with less than 5 CTC/7.5 mL, those individuals with non-elevated TIMP-1 had a median PFS of 12.3 months compared with only 4.4 months with elevated TIMP-1 levels. OS was 21.4 months with non-elevated but only 12.0 months with elevated TIMP-1. When five or more CTC were detected, patients with non-elevated TIMP-1 concentrations had a median PFS of 9.5 months vs. 8.5 months with elevated levels. The OS in the group with five or more CTC non-elevated TIMP-1 was 15.5 months vs. 12.0 months with elevated TIMP. No difference in OS was observed between patients with elevated TIMP-1 irrespective of CTC detection (Figure 2 and Table 4).

**Table 4:** Mean survival based on CTC positivity, TIMP-1 and CAIX levels

	PFS in months	*P *value	OS in months	*P *value
CTC negative		< 0.01^1^		< 0.01^1^
TIMP-1				
Non elevated	12.3(10.6-14.1)	< 0.01^2^	21.4 (20.3-22.6)	< 0.01
Elevated	4.4 (2.8-6.0)		12.0 (8.2-15.7)	
CTC positive				
TIMP-1				
Non elevated	9.5 (7.6-11.4)	0.81	15.5 (13.3-17.7)	0.151
Elevated	8.5 (6.2-10.8)		12.0 (9.4-14.6)	
CTC negative		< 0.01^1^		< 0.01^1^
CAIX				
Non elevated	11.6 (9.8-13.5)	0.06	21.3 (20.0-22.6)	< 0.01
Elevated	8.1 (5.6-10.6)		15.9 (12.8-19.0)	
CTC positive				
CAIX				
Non elevated	11.0(8.7-13.1)	< 0.05	16.2 (14.0-18.4)	< 0.05
Elevated	7.1(5.2-9.0)		11.8 (9.5-14.1)	

*^1^P value *for comparing differences between all four groups, ^2^comparing survival differences for comparing marker positivity within CTC negative and positive patients

CAIX, carbonic anhydrase IX; CTC, circulating tumor cell; OS, overall survival; PFS, progression-free survival; TIMP, tissue inhibitor of metalloproteinase.

**Figure 3:**
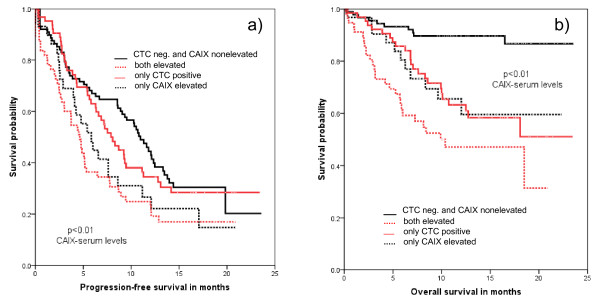
**Correlation between CTC detection, CAIX and patient outcome**. **(a) **Progression-free survival. **(b) **Overall survival of metastatic breast cancer patients based on CTC positivity (≥ 5 CTC) and CAIX serum levels. CAIX, carbonic anhydrase IX; CTC, circulating tumor cell.

Among patients with less than five CTC, those individuals with non-elevated CAIX had a median PFS of 11.6 months compared with 8.1 months with elevated CAIX. OS was 21.3 months with non-elevated and 15.9 months with elevated CAIX. When five or more CTC were detected, patients with non-elevated CAIX had a median PFS of 11.0 months versus 8.5 months with elevated levels. The OS in the CTC group with less than five CTC and non-elevated CAIX was 21.3 months vs. 15.9 months with elevated CAIX. In patients with five or more CTC, OS was 16.2 months with non-elevated and 11.8 months with elevated CAIX (Figure 3 and Table 4).

### Multivariate analysis

To evaluate the additional prognostic value of CAIX and TIMP-1, a multivariate analysis was performed for OS and PFS including the clinicopathological factors shown. For OS, only CTC, line of therapy and CAIX revealed to be independent prognostic factors. For PFS, only estrogen receptor, number of metastatic sites, and line of therapy were independent factors. Results of univariate and multivariate correlations are summarized in Table 5.

**Table 5:** Univariate and multivariate anlaysis for PFS and OS

	PFS Univariate *P *value	Multivariate *P *value	HR	95% CI	OS Univariate *P *value	Multivariate *P *value	HR	95% CI
**CAIX-levels** Elevated vs. non elevated	< 0.01	0.06	1.38	0.99 - 1.92	***< 0.01***	***.04***	***1.68***	***1.02 - 2.77***
**TIMP levels** Elevated vs. non elevated	< 0.01	0.21	1.26	0.88 - 1.80	< 0.01	.09	1.55	0.93 - 2.58
**Therapy line** > 1^st ^line vs. 1^st ^line	***< 0.01***	***< 0.01***	***2.61***	***1.84 - 3.70***	***< 0.01***	***< 0.01***	***2.61***	***1.45 - 4.70***
**Grading** 3 vs. 1/2	0.49	-	-	-	0.41	-	-	-
**Menopusal status** Premeno vs. postmeno	0.06	-	-	-	0.49	-	-	-
**ER status** Negative vs. positive	***0.03***	***0.01***	***1.51***	***1.90 - 2.10***	0.21	-	-	-
**PR status** Negative vs. positive	0.07	-	-	-	0.57	-	-	-
**HER2 status** Negative vs. positive	0.17	-	-	-	0.40	-	-	-
**Number of metastatic sites** Multiple vs. single	**< 0.01**	***0.02***	***1.52***	***1.08 - 2.15***	< 0.01	0.27	1.37	0.78 - 2.40
**CTC count** ≥ 5 cells vs. < 5 cells	0.12	-	-	-	***< 0.01***	***< 0.01***	***2.48***	***1.46-4.36***

CAIX, carbonic anhydrase IX; CI, confidence interval; CTC, circulating tumor cell; ER, estrogen receptor; HER, human epidermal growth factor receptor; HR, hazard ratio; OS, overall survival; PFS, progression-free survival; PR, progesterone receptor; TIMP, tissue inhibitor of metalloproteinase.

## Discussion

Insights into the biology of the metastatic potential of breast cancer cells are of relevance for several reasons. One clinical aspect is the identification of patients with more aggressive tumors that might benefit from more intense therapy. Another important reason is the need for an improved understanding of mechanisms leading to hematogenous tumor cell dissemination. This is of potential consequence for the development of new therapeutic approaches. Blood is often preferred over tumor tissue as it is easy to obtain. Also, repeated sampling is possible, which allows the use of markers for monitoring patients during the course of their disease. Thus, we examined the two serum factors TIMP-1 and CAIX and also investigated a correlation to the presence of CTC, which is a "real time" parameter of tumor cell dissemination.

Protein levels of TIMP-1 in tumor tissue are associated with prognosis and therapy response in patients with primary breast cancer [16-18,28] and with response to chemotherapy [29]. Schrohl and colleagues were able to show that elevated primary tumor levels for TIMP-1 also have a negative impact in the metastatic situation indicating that TIMP-1 has a general impact on tumor biology [29]. In an experimental study, also a role of TIMP-1 for chemoresistance was described [30]. It was recently shown in a prospective study that levels of TIMP-1 in plasma and serum obtained preoperatively from patients with primary breast cancer are associated with prognosis [31] whereas plasma levels at the time of primary surgery are not correlated with tissue concentrations [32]. Our results support the idea that also serum TIMP-1 levels reflect an enhanced ability of tumor tissues (including metastatic sites) to release cells into the circulation. In our patient cohort, higher TIMP-1 levels are associated with shorter PFS and OS in univariate analysis, supporting the biologic relevance of this factor. However, we cannot provide experimental evidence for a direct role of TIMP-1 in the release of CTC into the circulation. Rather than being involved in tumor cell release, TIMP-1 might stabilize released cells by inhibiting apoptosis through activation of survival pathways either by inhibition of MMPs or independently of MMPs. Furthermore, TIMP-1 protects tumor cells from chemotherapy-induced apoptosis [10-12]. In addition, TIMP-1 is expressed in a variety of cell types, including tumor cells and stromal cells and detectable in most tissues and in body fluids. It is also expressed by monocytes and macrophages and all these cells might contribute to high TIMP-1 concentrations in tumor tissue. Transcriptional analysis in colorectal cancer revealed that the expression of TIMP-1 in fibroblasts is even higher than that in tumor cells [33]. TIMP-1 protein expression was also high in stromal cells closest to tumor areas [34]. Therefore, secreted TIMP-1 in serum might be derived from different cell types (metastatic tumor cells, surrounding stromal cells, infiltrating macrophages, monocytes and others) making analysis of rare single cells for TIMP-1 difficult. Moreover, CTC were detected with the CellSearch system and the only free fluorescence channel for further characterization of CTC already was occupied for HER2 detection in our study. Furthermore, tumor tissue from metastatic sites was not available for further analysis in most cases. Therefore, it was not possible to directly examine the expression of TIMP-1 and/or CAIX in the tumor tissue or on CTC.

The fact that for TIMP-1 a relevant difference in PFS was observed only in patients with less than five CTC and the OS difference between patients with elevated and non-elevated TIMP-1 was also larger in patients with low CTC numbers could indicate that serum TIMP-1 is especially relevant in patients without the detection of elevated CTC counts. This might open a clinical perspective for this marker also in the context of CTC measurements because even in metastatic breast cancer a relevant portion has CTC counts below the widely established cut off of five or more CTC in 7.5 mL blood. In patients with elevated TIMP-1, OS did not differ between patients with CTC findings above and below the cut off. Therefore, TIMP-1 does not seem to add prognostic relevance in CTC-positive patients. Currently, we do not have an explanation for the observation that patients with elevated TIMP-1 and less than five CTC/7.5 ml had the shortest PFS (Figure 2 and Table 4). In addition, it is not possible to exclude that at least some patients with elevated TIMP-1 levels who are detected to be "CTC-negative" in the CellSearch assay represent a subpopulation of patients with still undetectable CTC that have lost their epithelial characteristics in the course of epithelial-mesenchymal transition [35].

A generally accepted normal level of TIMP-1 serum concentration was not defined so far. Our study was not designed to validate cut-off values for these markers. Lipton et al. applied a cut-off of 454 ng/mL (95% of the control group) when they used control group of 49 healthy postmenopausal women to derive the serum TIMP-1 cut-off with the same assay as used in our study [7].

Altered glycolysis is a main metabolic feature of malignant cells. Combined with decreased oxidative phosphorylation it can result in acidification of the extracellular space [36,37]. Transcription factors of the glycolytic pathway also influence cell proliferation and differentiation; disordered glycolysis and acidic milieu were therefore proposed to play a major role in the complex multistep process of carcinogenesis [37-41]. One of the enzymes contributing to acidification of the extracellular space is CAIX. It is a transmembrane zinc enzyme catalyzing the hydration of carbon dioxide [42]. CAIX is strongly induced by hypoxia via activation of transcriptional factors such as HIF-1 [43]. CAIX is overexpressed in a variety of solid tumors with different results for its potential role in gynecologic cancers and breast cancer [44-48]. The inhibition of this enzyme is a potiential therapeutic approach [49-51]. In renal cell cancer, overexpression of CAIX is common and the possible role of CAIX targeting antibodies (WX-G250, Rencarex^®^) is currently being evaluated in phase III trials for this entity [52]. There is some information on serum values in renal cell cancer patients showing significantly higher values in patients with metastatic disease than in patients with localized cancer. Furthermore, renal cell cancer patients with high serum CAIX before surgery were at significantly higher risk for disease recurrence than those with low preoperative values [23]. The role of serum CAIX in breast cancer has not been determined. As hypoxia is postulated to be associated with hematogenous tumor cell dissemination in breast cancer [53], we examined this factor in correlation to the detection of CTC. It appears that CAIX alone has prognostic significance in both CTC-negative and CTC-positive groups and we provide to our knowledge the first evidence for a prognostic relevance of serum CAIX. This is of potential clinical relevance for the application of new therapeutic approaches inhibiting angiogenesis or directly CAIX.

Moreover, our findings show an association between elevated CAIX serum levels and the presence of CTC which also supports the experimental findings that indicate an association between hypoxia and release of tumor cells into the circulation. Our observations also could indicate that CAIX in contrast to TIMP-1 has prognostic relevance in patients with elevated and non-elevated CTC numbers (Figure 3 and Table 4). However, we cannot definitely explain the correlation we observed because the cellular origin of serum CAIX in our patients is not clear. Elevated levels of both CAIX and TIMP-1 were seen in 18% of patients (Figure 1; Table 2). However, in the overall cohort no relevant improvement for the prognostic information concerning PFS and OS was observed. This does not deliver the rationale for a combined use as prognostic markers.

The lack of correlation between CTC detection and PFS in multivariate analysis in our cohort might be due to different treatments, therapeutic settings (1^st ^line, 2^nd ^line, 3^rd ^line and more), metastatic sites, and by slightly different response monitoring according to the institutional standards of the participating Breast Cancer Centers. For example, in the currently most cited publication using the CellSearch System also applied in our study [3], 47% of patients were starting their first line of therapy whereas in our cohort the rate was only 38%. In addition, the use of targeted therapies (trastuzumab and lapatinib for HER2-positive and bevacizumab for HER2-negative patients) might change the prognostic relevance of CTC detection with respect to PFS as the end point. Similarly to other studies, we did not observe a correlation between CTC detection and estrogen receptor/progesterone receptor and HER2 status of the corresponding primary tumors [3]. The fact that we did not detect a significantly higher number of CTC-positive patients having bone metastasis compared with other sites of metastasis is not in line with some other findings, but might be explained by the relatively small number of patients only with bone metastasis (*n *= 35) enrolled in our patient study. Taken together, this lack of standardized treatment is a potential drawback of the study However, the strength of our study is the prospective and multicenter setting and the analysis of biomarkers blinded for clinical data.

## Conclusions

This study demonstrates with a prospective design that serum TIMP-1 and CAIX have prognostic impact in metastatic breast cancer and elevated serum levels of these invasion markers are associated with the presence of CTC-positivity. The mechanisms that lead to tumor cell dissemination from primary tumors or metastases have not been recognized in detail yet. To find out whether TIMP-1 and CAIX are involved in this process, for example by loosening cell adhesion, changing pH conditions or prolongation of CTC survival has to be further investigated in experimental model systems.

## Abbreviations

CAIX: carbonic anhydrase IX; CT: computed tomography; CTC: circulating tumor cell; ELISA: enzyme linked immunosorbent assay; HER2: human epidermal growth factor receptor 2; MMP: matrix metalloproteinases; OS: overall survival; PFS: progression-free survival; TIMP: tissue inhibitor of metalloproteinase 1.

## Competing interests

Wolfgang Janni, Brigitte Rack and Klaus Pantel have received educational grants from Veridex. Tanja Fehm has received unrestriceted research support from Adnagen. The other authors have no relevant competing interest to declare.

## Authors' contributions

VM and TF participated in the conception and design of the study on TIMP-1 and CAIX and drafted the paper; all authors participated in the acquisition of data and patient recruitment as well as in conception of the DETECT study, analysis and interpretation of data, revising the paper critically for important intellectual content and gave final approval of the version submitted. All authors read and approved the final manuscript.
